# Hypertension is a marker of the micro-epidemiologic transition in ageing HIV populations in Kenya, Uganda and Tanzania (AFRICOS, 2013–2023)

**DOI:** 10.1186/s12889-026-27241-3

**Published:** 2026-04-02

**Authors:** Denis Mayambala, Wandera Stephen Ojjambo, Charles Lwanga, Dan T Haydon, Bassirou Bonfoh, Sayoki G. Mfinanga, Hannah Kibuuka, Francis Sena Nuvey

**Affiliations:** 1 Department of Population Studies, School of Statistics and Planning, College of Business and Management Sciences, Makerere University, P.O. Box 7062, Kampala, Uganda; 2 School of Biodiversity, One Health and Veterinary Medicine, College of Medical, Veterinary and Life Sciences, University of Glasgow, Glasgow, G12 8QQ UK; 3 Centre Suisse de Recherches Scientifiques en Côte d’Ivoire, Abidjan, 1303 BP Côte d’Ivoire; 4 National Institute for Medical Research, Muhimbili Medical Research Centre, P.O. Box 3436, Dar es Salaam, Tanzania; 5 Makerere University Walter Reed Program (MUWRP), Kampala, Uganda; 6 Friedrich-Loeffler-Institut, Suedufer 10, 17493 Greifswald, Germany

**Keywords:** HIV, Hypertension, Mortality, Life-course, East Africa, Demography, AFRICOS

## Abstract

**Background:**

Although mortality among people living with HIV (PLWH) in sub-Saharan Africa has decreased with antiretroviral therapy (ART) scale-up, the demographic consequences remain underexamined.

**Methods:**

Using ten years of longitudinal data from the African Cohort Study (AFRICOS; 2013–2023) in Kenya, Tanzania, and Uganda, we estimated the association of hypertension with all-cause mortality among adults aged ≥ 40 years. We combined decremental life-table analysis with discrete-time logistic regression, applying lagged and exponentially weighted moving-average (EWMA) exposure models to capture cumulative risk.

**Results:**

At enrollment, 18.6% were hypertensive; 60.1% experienced hypertension during follow-up, and all-cause mortality was 6.4%. Life-table estimates showed cumulative excess mortality became statistically significant from year 7 onward, reaching 3.4% (95% CI: 1.3–5.6) by year 9 before plateauing. Excess mortality was largest among adults aged 50–59 years (4.9% [1.8-8.0]), men (6.0% [1.4–10.5] by year 8), and underweight participants (27.1% [5.4–48.8] by year 9). Participants with suppressed viral load exhibited significant excess throughout follow-up (5.5% [3.3–7.8] by year 9). Tanzania showed the earliest and most persistent excess (≈ 5% from year 2 onward). Among underweight participants, hypertensives had a 3.6-fold higher mortality rate than non-hypertensives (58.3 vs. 16.2 per 1000 PY; *p* = 0.009). Similarly, among those with suppressed HIV viral load, hypertension was associated with a 2.3-fold higher mortality rate (11.4 vs. 4.9 per 1000 PY; *p* = 0.025). In adjusted models, hypertension was associated with higher mortality under standard lag (aOR = 2.04; 1.10–3.80) and EWMA specifications (aOR = 3.25; 1.26–8.40 at α = 0.3; aOR = 2.51; 1.16–5.44 at α = 0.7). Mortality odds were higher among participants aged ≥ 60 years (aOR = 2.23–2.40) and those with high viral load (aOR = 2.36–2.44), while overweight and obese participants had substantially lower odds of death (aOR = 0.29; 0.10–0.85 and aOR = 0.14; 0.04–0.56).

**Conclusions:**

These findings highlight a demographic transformation of the HIV epidemic in East Africa, where mortality among PLWH increasingly reflects chronic disease influence. Hypertension has become a key driver of excess mortality and a demographic indicator of the region’s compressed health transition.

## Background

 Over the past two decades, lower- and middle-income countries (LMICs) have undergone a profound epidemiological and demographic transition. This is marked by a shift away from communicable, maternal, neonatal, and nutritional causes toward non-communicable diseases (NCDs) as the dominant sources of mortality and disability. NCDs now account for approximately 71% of global deaths, including nearly 18 million cardiovascular deaths annually [1]. sub-Saharan Africa (SSA) is experiencing a rapid rise in NCD-related disability and mortality [2] driven by urbanization, dietary change, and declining physical activity [3].

Within this broader transition, HIV remains endemic in SSA, and it currently comprises about two-thirds of the worldwide population of people living with HIV (PLWH) [4, 5]. The large-scale expansion of antiretroviral therapy (ART) has fundamentally reshaped mortality patterns among PLWH. Cohorts once characterized by high levels of premature adult mortality are now surviving into older ages, producing a marked upward shift in the age schedule of HIV mortality [6, 7]. In Uganda’s Rakai cohort, partial life expectancy before age 50 increased by 7.1 years for women and 5.2 years for men within a single treatment generation [8]. As a result, morbidity and mortality among PLWH increasingly reflect the same epidemiological forces driving population-level NCD transitions, rather than HIV-specific opportunistic disease.

As a consequence, PLWH’s present-day population structure is demographically distinct: it is larger, older, and increasingly characterised by the co-occurrence of chronic conditions. Globally, the proportion of PLWH aged 50 years and older nearly doubled between 2000 and 2016, with almost 80% residing in LMICs and SSA accounting for the largest absolute numbers [9]. Although PLWH remain a numerical minority within older adult populations, modelling studies consistently show that they are disproportionately represented among individuals experiencing NCD incidence and multimorbidity, particularly combinations involving HIV, hypertension, and diabetes in high-prevalence settings [10, 11].

Those patterns are consequential in SSA, which continues to host nearly two-thirds of the global PLWH population [4]. Longitudinal and simulation-based analyses further suggest that, even as survival improves, PLWH accumulate chronic conditions earlier and spend fewer years free of multimorbidity, positioning this population as a critical lens through which to examine multi-disease accumulation under conditions of constrained health system capacity [5, 10]. The main problem of the existence of NCDs is the emergence of multimorbidity [12], especially the combination of HIV, hypertension, and diabetes. Meta-analyses estimate hypertension in roughly one-in-five PLWH in SSA, with disproportionate clustering among older adults, men, and those with higher CD4 counts on long-term ART [4, 10].

The rise of hypertension is a quiet but consequential demographic force. Its prevalence now rivals that of any major infectious comorbidity among PLWH, ranging from 18 to 27 per cent in East Africa [4, 13]. Hypertension manifests earlier in the life course, often beginning in the forties (40s) and reflecting etiologies that extend beyond pharmacologic side effects to include prolonged immune activation, nutritional transition, and psychosocial stress [14]. As AIDS-related mortality declines, cardiovascular and hypertensive causes increasingly occupy the mortality schedule of treated HIV populations [15, 16]. This shift exemplifies what Kuate Defo described as Africa’s dual burden of disease: the coexistence of infectious and chronic degeneration within the same cohorts [17].

This paper introduces the concept of a micro-epidemiologic transition, which is a shift from infection-dominated to chronic, circulatory mortality occurring within ART-treated HIV cohorts rather than across whole societies. The central question is structural rather than purely clinical: *To what extent do chronic vascular processes now account for mortality among ageing PLWH*,* and how do they reshape cohort survival patterns?* Conventional HIV mortality models continue to prioritise AIDS-specific causes of death, thereby understating the contribution of chronic comorbidities to all-cause mortality. Although life-table decomposition and decrement analysis offer robust tools for quantifying cause-specific contributions to survival change [18]. These methods have rarely been applied to African HIV cohorts now entering older adulthood. This gap limits our ability to measure the population-level consequences of the health transition already underway.

Theoretically, this transition can be understood through the lenses of cumulative disadvantage and the life course [19, 20]. Early HIV infection, delayed treatment initiation, and chronic psychosocial stress accumulate physiological wear, which McEwen termed allostatic load, manifesting as hypertension later in life [21]. Thus, hypertension becomes not only a clinical risk factor but as a demographic signal of inequality embedded across decades of exposure [22]. Empirical evidence underscores the stakes of this transition, where uncontrolled hypertension has been shown to double all-cause mortality among PLWH in South Africa [16], while cardiovascular deaths are rising within Tanzanian ART programmes [15].

Despite evidence linking hypertension to mortality among ageing PLWH in sub-Saharan Africa, existing studies have relied on clinical effect measures that do not capture population-level burden. Using longitudinal data from the African Cohort Study (AFRICOS, 2013–2023), we aimed to quantify the demographic association between hypertension and mortality among PLWH aged 40 years and older in Kenya, Uganda, and Tanzania. We hypothesized that hypertension confers a progressively widening survival disadvantage over time. This analysis introduces the concept of a *micro-epidemiologic transition* to frame the shift from infection-dominated to chronic disease mortality within ART-treated HIV cohorts in the region.

## Data and methods

### Study design and setting

This study used longitudinal data from the African Cohort Study (AFRICOS), an ongoing prospective cohort established in 2013 at 12 clinics across five PEPFAR-supported programmes, including sites in Kayunga (Uganda), South Rift Valley and Kisumu West (Kenya), Mbeya (Tanzania), and Lagos and Abuja (Nigeria) [13]. For this analysis, we restricted ourselves to participants from the East African sites (Kenya, Uganda, and Tanzania) because these sites had the longest follow-up, the largest sample of ageing PLWH ensuring regional comparability. This design is well-suited to our research question: it provides a decade of repeated blood pressure and biomarker measurements, systematic mortality ascertainment, and a large, well-characterized sample of ageing PLWH in East Africa, where hypertension burden is rising but demographic impact remains unquantified.

### Sampling and recruitment

At each site, people living with HIV (PLWH) were recruited from randomized lists of current PEPFAR clinic patients and those with new HIV diagnoses. Enrolees were encouraged to bring partners for testing and recruitment. People living without HIV were recruited from community members accessing HIV testing services, with a small subset recruited from prior research studies.

### Sample size considerations

The analytic sample included 1,169 participants at enrolment year 2013, 12,462 person-periods of follow-up. With 60.1% of participants contributing hypertensive person-time and an observed mortality rate of 6.4%. The study had approximately 86% power to detect a minimum hazard ratio of 1.4 for all-cause mortality comparing hypertensive to non-hypertensive person-time, assuming a two-sided α = 0.05 and 10% loss to follow-up.

### Inclusion and exclusion criteria

Participants were eligible for AFRICOS enrollment if they were aged ≥ 15 years, intended to be long-term residents of the area, willing to provide contact information, able to provide written informed consent, and able to understand English or the local language. Individuals were excluded if they were pregnant at enrollment. For this analysis, we applied additional criteria to address our research question on ageing HIV populations. We included participants who were aged 40 years or older at enrollment, had confirmed HIV-positive status, and contributed a follow-up visit between 2013 and 2023. We excluded participants with missing blood pressure measurements at all visits.

### Study procedures

At enrollment, all participants completed a medical history, physical examination, and demographic questionnaire, and underwent phlebotomy. PLWH underwent confirmatory HIV rapid diagnostic testing, CD4 count, and HIV viral load testing [23]. Study visits occurred every six months, at which participants provided updated medical history, completed physical examinations, and underwent laboratory assessments. Study clinicians performed medical record reviews and extracted ART start dates and regimens at every visit.

### Outcome

The outcome was all-cause mortality observed during follow-up in a given calendar year. Deaths were confirmed through clinic and community tracing systems and coded at the next scheduled visit. Time was measured in half-year intervals corresponding to the AFRICOS visit schedule.

### Exposure

Hypertension, the principal exposure, is defined as systolic blood pressure ≥ 140 mm Hg, diastolic ≥ 90 mm Hg, determined via participant chart review at any visit.

### Covariates

Covariates for adjusted models were selected based on prior literature and theoretical considerations of factors known to influence both hypertension and mortality among ageing people living with HIV. BMI was calculated as weight (kg) divided by height (m²) using measured height and weight. Categories were defined as: underweight (< 18.5), normal weight (18.5–24.9), overweight (25.0–29.9), and obese (≥ 30). Viral load was categorized using standard WHO clinical thresholds as: 1 = suppressed (< 50 copies/mL), 2 = low-level viremia (50–999 copies/mL), and 3 = high (≥ 1000 copies/mL). BMI, and viral load were treated as time-varying.

### Statistical analysis

We described the study population at enrollment using frequencies and percentages. To examine bivariate associations, we used chi-square tests for categorical variables and Poisson regression with an offset for log person-years for incidence rates, reporting rate differences with 95% confidence intervals. We then, constructed decremental Life tables to provide an intuitive, population-level description of cumulative mortality differences. We estimated the interval probability of death (qₓ) separately for hypertensive and non-hypertensive individuals. For each yearly interval, qₓ was calculated as:

where lₓ represents the number of individuals at risk at the beginning of the interval and dₓ represents the number of deaths occurring within that interval and we assumed it to occur nearest to mid-year. To stabilize estimates, we applied LOESS smoothing (degree 1, span = 0.4) to the raw qₓ values, borrowing information from neighboring intervals to produce more reliable estimates. From the smoothed mortality probabilities (q̂ₓ), survival probabilities were derived as:

$$q_\mathrm{x}\;=\;d_\mathrm{x}/l_\mathrm{x}$$

The number of survivors at the start of each subsequent interval was computed recursively as:

$$p_\mathrm{x}\;=\;1\;-\;\hat{q}_\mathrm{x}$$

Deaths in each interval were re-calculated as:

$$l_\mathrm{x+1}\;=\;l_\mathrm{x}\;\times\;p_\mathrm{x},\;with\;l_1\;=\;1.$$

Person-years lived within each interval were estimated as:

$$L\mathrm{x}\;=\;l\mathrm{x}\;-\;0.5\;d_\mathrm{x}$$

assuming deaths occur uniformly within intervals. Cumulative mortality up to interval x was calculated as:

$$\mathrm{M}_\mathrm{x}\;=\;1\;-\;\prod{_{i=1}}^\mathrm{x}\;p_\mathrm{i}.$$

Standard errors for cumulative mortality were approximated using a delta method that propagated uncertainty from the smoothed interval mortality probabilities through the life table calculations. The variance of cumulative mortality was computed as

$$\:\widehat{Var}\left({M}_{x}\right)={\sum\:}_{i=1}^{x}{\left(\frac{\partial\:{M}_{x}}{\partial\:{q}_{i}}\right)}^{2}\widehat{Var}\left({\widehat{q}}_{i}\right),$$

assuming independence between interval and 95% confidence intervals were constructed as

$$\:{\widehat{M}}_{x}\pm\:1.96\times\:\widehat{SE}\left({\widehat{M}}_{x}\right).$$

The difference $\:\varDelta\:{q}_{\mathrm{x}}={{q}_{\mathrm{x}}}^{HTN}-{{q}_{\mathrm{x}}}^{non-HTN}$​ represented the excess probability of dying associated with hypertension.

Secondly, to verify that the observed $\:\varDelta\:{q}_{\mathrm{x}}\:$were not driven by measured confounders, we fitted discrete-time logistic regression models. Covariates were selected a priori based on established associations with both hypertension and mortality in the literature. No automated variable selection procedures were used. The discrete-time logistic regression modeled the probability of death in each interval conditional on survival up to that interval, accounting for the longitudinal structure of the data and flexibly accommodating time-varying covariates [24–26]. The regression results serve to confirm that the hypertension-mortality association persists after adjusting for age, sex, BMI, viral load, and country;

$$\begin{aligned}\:\mathrm{log}\left(\frac{\left(P\right({Death}_{it}=1)}{1-P\left({Death}_{it}=1\right)}\right)&={\beta\:}_{0}+{\beta\:}_{1}{EMA}_{{HTN}_{i\left(t-1\right)}}\\&+{\beta\:}_{2}{X}_{i\left(t-1\right)}+\gamma\:t\end{aligned}$$

where $\:{EMA}_{{HTN}_{i\left(t-1\right)}}$represented the lagged exponential moving average of hypertension status for individual i at time $\:t-1$, $\:{X}_{i\left(t-1\right)}$ is a vector of lagged covariates including country, age category, sex, BMI category, and viral load category,$\:\:{\beta\:}_{0}$​ is the intercept, $\:\beta\:2$​ is the vector of coefficients for confounders, and $\:\gamma\:t$ represented the visit-specific fixed effects, accounting for unobserved heterogeneity across time points.

We compared three exposure specifications: (1) standard lagged hypertension status (hypertension at the previous visit) to establish temporal precedence between hypertension exposure and mortality, all time-varying covariates were lagged by one visit [27]. Secondary, exponentially weighted moving average (EWMA) with smoothing parameters α = 0.3 and α = 0.7. We constructed EWMA to reflect cumulative exposure over the life course. The EWMA at time t was calculated recursively as

     

$$\:EMA_t\:=\:\alpha\:\:\times\:\:X_t\:+\:(1-\alpha\:)\:\times\:\:EMA_{t-1},$$

where $\:X_\mathrm{t}$ is hypertension status at the current visit. This approach assigned greater weight to recent observations while retaining information on prior exposure, with α = 0.3 giving more weight to historical values and α = 0.7 emphasizing recent exposure [28]. All exposure variables were lagged by one visit to ensure temporal precedence.

Standard errors were clustered at the individual level to account for repeated measures [29]. Results are presented as adjusted odds ratios (aOR) with 95% confidence intervals. These two approaches are complementary: life tables document demographic risk accumulation, while regression models isolate independent effects after controlling for confounders. Model fit was compared using Akaike Information Criterion (AIC) and Bayesian Information Criterion (BIC). We also tested for interactions between hypertension measures and time to assess whether mortality differentials changed over follow-up. All analyses were conducted using R (version 4.2.1) with the survival and tidyverse packages and (STATA version 18).

## Results

### Descriptive characteristics

Table 1 summarizes the demographic and clinical composition of the analytic cohort (*n* = 1,169), representing a maturing population of PLWH transitioning into mid- and later life. Participants were predominantly aged 40–49 years (56.7%), male (51.2%) median (IQR) time to event of 7.1 (4.9–8.6), and resident in Kenya (59.7%) median (IQR) time to event of 7.5 (6.4–8.7). Hypertension was present in nearly one-fifth of participants at enrollment (18.6%) and affected more than half of the cohort (60.1%), median time from study entry to first hypertensive visit was 2.1 years (IQR: 0.8–4.3 years). All participants were already on ART (median ART duration at enrolment: 3.4 years). Overall mortality during follow-up was 6.4%, consistent with a cohort in which infectious mortality has largely receded but not disappeared. The prevalence of hypertension increased from 13.6% among those aged 40–49 years to 32.3% among those 60 years and older (*p* < 0.001), while mortality followed a similar gradient from 5.7% to 12.5% (*p* = 0.038). Men and women displayed near-equal hypertension prevalence (18.0–19.7%, *p* = 0.496), yet men’s mortality remained higher (7.9% vs. 4.9%, *p* = 0.052).

**Table 1:** Characteristics of the study population at enrollment and distribution of hypertension and mortality (AFRICOS 2013–2023)

Variable	*n* (%)	Hypertensive *n* (row%)	*p*-value (hypertension)	Dead *n* (row%)	*p*-value Death
Overall Total	1169 (100)	221 (18.9)		75 (6.4)	
Sex					
Male	596 (51)	107 (18)	0.496	47 (7.9)	0.052
Female	569 (48.7)	112 (19.7)		28 (4.9)	
missing	4 (0.3)	2 (50)		0 (0)	
Age group (years)					
40–49	720 (61.6)	98 (13.6)	< 0.001	41 (5.7)	0.038
50–59	349 (29.9)	90 (25.8)		22 (6.3)	
60+	96 (8.2)	31 (32.3)		12 (12.5)	
Missing	4 (0.3)	2 (50)			
BMI category (kg/m²)					
underweight	142 (12.1)	18 (12.7)	< 0.001	19 (13.4)	0.001
Normal	717 (61.3)	101 (14.1)		44 (6.1)	
Overweight	210 (18)	65 (31)		10 (4.8)	
Obese	100 (8.6)	37 (37)		2 (2)	
Viral load status (copies/mL)					
Suppressed	694 (59.4)	146 (21)	0.019	30 (4.3)	0.001
low-level viremia	132 (11.3)	27 (20.5)		10 (7.6)	
High viral load	343 (29.3)	48 (14)		35 (10.2)	
Country of residence					
Kenya	698 (59.7)	118 (16.9)	< 0.001	44 (6.3)	0.091
Tanzania	256 (21.9)	82 (32)		11 (4.3)	
Uganda	215 (18.4)	21 (9.8)		20 (9.3)	

Data presented as n (%). Row percentages shown for hypertensive and dead columns. *P*-values from chi-square tests. *p* < 0.05, *BMI* body mass index. Percentages for categories with small sample sizes (e.g., missing data) should be interpreted with caution

Hypertension prevalence increased steadily with body mass from 12.7% among the underweight to 37% among the obese (*p* < 0.001). In contrast, mortality declined across the same gradient, from 13.4% among the underweight to 2.0% among the obese (*p* < 0.001). Viral suppression status further stratified survival outcomes. Although overall suppression was high (59.4.0%), mortality differed sharply by viral load, with significantly lower mortality among suppressed individuals (4.3%) compared with (10.2%) among those with high viremia (*p* < 0.001). Hypertension was more common among the suppressed (21.0%) than those with high viral load (14.0%, *p* = 0.019) within the cohort. Geographic variations observed mirror structural differences in health-system performance. Kenya, contributing the largest share of participants, exhibited intermediate hypertension prevalence (16.9%) and mortality (6.3%). Tanzania showed the highest hypertension prevalence (32.0%) but lowest mortality (4.3%), whereas Uganda showed the reverse pattern, with lower hypertension (9.8%) but higher mortality (9.3%).

Table 2 presents mortality incidence rates per 1000 person-years comparing hypertensive and non-hypertensive participants across demographic and clinical subgroups. These rates provide a summary measure of the overall mortality burden associated with hypertension over the entire follow-up period. Among underweight participants, hypertensives had a 3.6-fold higher mortality rate than non-hypertensives (58.3 vs. 16.2 per 1000 PY; *p* = 0.009). Similarly, among those with suppressed HIV viral load, hypertension was associated with a 2.3-fold higher mortality rate (11.4 vs. 4.9 per 1000 PY; *p* = 0.025). In Tanzania, hypertensives had a 3.1-fold higher mortality rate compared to non-hypertensives (16.3 vs. 5.2 per 1000 PY; *p* = 0.061), approaching statistical significance. No other subgroups showed statistically significant differences.

**Table 2:** Mortality rates by hypertension status across participant characteristics

Characteristic	Non-hypertensive *n*	Deaths	Rate per 1000 PY (95% CI)	Hypertensive *n*	Deaths	Rate per 1000 PY (95% CI)	*p*-value
Age group (years)							
40–49	595	34	8.4 (5.6–11.2)	91	7	11.6 (3.0–20.2)	0.430
50–59	251	14	8.1 (3.9–12.4)	85	8	14.0 (4.3–23.7)	0.222
≥ 60	62	9	22.0 (7.6–36.4)	28	3	16.8 (0.0–35.9)	0.687
Sex							
Male	468	35	11.1 (7.4–14.8)	100	12	18.8 (8.2–29.4)	0.115
Female	437	22	7.3 (4.2–10.3)	102	6	8.6 (1.7–15.4)	0.725
Body mass index (kg/m²)							
Underweight	122	13	16.2 (7.4–24.9)	17	6	58.3 (11.6–104.9)	**0.009**
Normal	588	37	9.2 (6.3–12.2)	95	7	10.9 (2.8–19.0)	0.683
Overweight	138	6	6.4 (1.3–11.6)	61	4	10.5 (0.2–20.8)	0.446
Obese	61	1	2.3 (0.0–6.7)	33	1	4.2 (0.0–12.6)	0.659
HIV viral load status (copies/mL)							
Suppressed	533	19	4.9 (2.7–7.1)	139	11	11.4 (4.7–18.2)	**0.025**
Low-level viremia	99	10	16.7 (6.4–27.1)	24	0	0.0 (0.0–0.0)	0.993
High viral load	277	28	16.4 (10.3–22.4)	43	7	30.4 (7.9–53.0)	0.142
Country of residence							
Kenya	561	35	8.8 (5.9–11.8)	114	9	10.6 (3.7–17.5)	0.630
Tanzania	162	5	5.2 (0.6–9.8)	71	6	16.3 (3.3–29.3)	**0.061**
Uganda	186	17	13.4 (7.0–19.8)	21	3	21.5 (0.0–45.8)	0.452

*PY*person-years, *CI* confidence interval. Rates are per 1000 person-years. *P*-values from Poisson regression comparing incidence rates between hypertensive and non-hypertensive groups. Values in bold indicates statistical significance (*p* < 0.05)

### Life-table analysis of excess mortality

Table 3 provides cumulative mortality year-by-year, illustrating how the mortality difference between groups evolves over time revealing the temporal dynamics of the association between hypertension and mortality. Overall, excess mortality became statistically significant from year 7 onward, reaching 3.4% (95% CI: 1.3–5.6%) by year 9 before plateauing. By age group, statistically significant differences emerged only among participants aged 50–59 years, where excess mortality reached 4.9% (1.8-8.0%) by year 9 and persisted through follow-up. Estimates for other age groups were imprecise with confidence intervals spanning zero. Men showed significant excess mortality from year 7 onward, peaking at 6.0% (1.4–10.5%) by year 8. Women demonstrated a modest but significant excess only in later years (1.8% [0.3–3.4%] by year 10). Underweight participants had the largest and most sustained excess, reaching statistical significance from year 7 (21.2% [0.3–42.1%]) and peaking at 27.1% (5.4–48.8%) by year 9. Overweight participants showed significant excess only in later years (5.2% [1.9–8.5%] by year 10). No significant differences were observed among normal-weight or obese participants. Participants with suppressed HIV viral load demonstrated consistent, statistically significant excess mortality throughout follow-up, increasing from 1.0% (0.3–1.7) in year 1 to 5.5% (3.3–7.8%) by year 9. Those with high viral load showed elevated but non-significant estimates, while participants with low-level viremia exhibited significantly lower mortality among hypertensives in later years (-11.1% [-14.7 to -7.6%] by year 8). Tanzania showed the earliest and most persistent significant excess, reaching 6.0% (1.0-11.1%) by year 2 and remaining stable at approximately 5% thereafter. Kenya demonstrated modest but significant excess only in later years (2.4% [0.1–4.7%] by year 10). Estimates for Uganda were consistently non-significant.

**Table 3:** Cumulative excess mortality (Δqₓ) by demographic and clinical subgroup, AFRICOS 2013–2023

Variable	1	2	3	4	5	6	7	8	9	10	11	
Hypertension status	0.0061 (-0.01-0.02)	0.0100 (-0.01-0.03)	0.0098 (-0.01-0.03)	0.0058 (-0.01-0.02)	0.0072 (-0.01-0.02)	0.0124 (-0.00-0.03)	**0.0210 (0.00-0.04)**	**0.0293 (0.01–0.05)**	**0.0343 (0.01–0.06)**	**0.0334 (0.01–0.06)**	**0.0326 (0.01–0.05)**	
Age group												
40–49	0.0062 (-0.02-0.03)	0.0149 (-0.01-0.04)	0.0163 (-0.01-0.04)	0.0093 (-0.02-0.04)	0.0059 (-0.02-0.03)	0.0029 (-0.02-0.03)	0.0047 (-0.02-0.03)	0.0154 (-0.01-0.05)	0.0282 (-0.01-0.06)	0.0331 (-0.00-0.07)	0.0331 (-0.00-0.07)	
50–59	0.0063 (-0.01-0.02)	0.0082 (-0.01-0.03)	0.0121 (-0.01-0.04)	0.0172 (-0.01-0.04)	0.0179 (-0.01-0.04)	0.0211 (-0.00-0.05)	**0.0327 (0.00-0.06)**	**0.0445 (0.01–0.08)**	**0.0494 (0.02–0.08)**	**0.0494 (0.02–0.08)**	**0.0494 (0.02–0.08)**	
60+	0.0189 (-0.03-0.06)	-0.0021 (-0.05-0.05)	-0.0309 (-0.09-0.03)	-0.0598 (-0.12-0.00)	-0.0765 (-0.14-0.01)	0.0108 (-0.12-0.14)	0.0033 (-0.13-0.14)	-0.0077 (-0.14-0.13)	-0.0505 (-0.18-0.08)	-0.1009 (-0.26-0.06)	-0.1274 (-0.28-0.03)	
Sex												
Female	-0.0054 (-0.01-0.00)	-0.0023 (-0.02-0.01)	-0.0008 (-0.02-0.01)	-0.0041 (-0.02-0.01)	-0.0043 (-0.02-0.01)	-0.0021 (-0.02-0.01)	-0.0019 (-0.02-0.01)	0.0027 (-0.01-0.02)	0.0121 (-0.00-0.03)	**0.0183 (0.00-0.03)**	**0.0183 (0.00-0.03)**	
Male	0.0187 (-0.01-0.05)	0.0240 (-0.01-0.06)	0.0223 (-0.01-0.05)	0.0177 (-0.01-0.05)	0.0210 (-0.01-0.05)	0.0298 (-0.00-0.06)	**0.0478 (0.00-0.09)**	**0.0596 (0.01–0.11)**	**0.0596 (0.01–0.11)**	**0.0511 (0.00-0.10)**	**0.0495 (0.00-0.10)**	
BMI category (kg/m²)												
Normal	-0.0038 (-0.01-0.00)	-0.0073 (-0.02-0.00)	-0.0098 (-0.02-0.00)	-0.0121 (-0.02-0.00)	-0.0071 (-0.02-0.00)	0.0027 (-0.01-0.02)	0.0156 (-0.01-0.04)	0.0209 (-0.00-0.05)	0.0184 (-0.01-0.04)	0.0116 (-0.01-0.04)	0.0103 (-0.02-0.04)	
Overweight	0.0032 (-0.01-0.02)	0.0084 (-0.01-0.03)	0.0098 (-0.01-0.03)	0.0072 (-0.02-0.03)	0.0050 (-0.02-0.03)	0.0050 (-0.02-0.03)	0.0050 (-0.02-0.03)	0.0186 (-0.01-0.04)	**0.0386 (0.01–0.07)**	**0.0517 (0.02–0.08)**	**0.0517 (0.02–0.08)**	
Underweight	0.1224 (-0.05-0.29)	0.1630 (-0.04-0.37)	0.1624 (-0.04-0.37)	0.1485 (-0.06-0.35)	0.1560 (-0.05-0.36)	0.1737 (-0.04-0.38)	**0.2122 (0.00-0.42)**	**0.2482 (0.03–0.46)**	**0.2710 (0.05–0.49)**	**0.2710 (0.05–0.49)**	**0.2710 (0.05–0.49)**	
Obese	-0.0159 (-0.03-0.00)	0.0154 (-0.03-0.06)	0.0154 (-0.03-0.06)	0.0154 (-0.03-0.06)	0.0154 (-0.03-0.06)	0.0154 (-0.03-0.06)	0.0154 (-0.03-0.06)	0.0154 (-0.03-0.06)	0.0154 (-0.03-0.06)	0.0154 (-0.03-0.06)	0.0154 (-0.03-0.06)	
Viral load status (copies/mL)												
Suppressed	**0.0099 (0.00-0.02)**	**0.0157 (0.01–0.02)**	**0.0176 (0.01–0.03)**	**0.0168 (0.01–0.03)**	**0.0198 (0.01–0.03)**	**0.0270 (0.02–0.04)**	**0.0385 (0.02–0.06)**	**0.0498 (0.03–0.07)**	**0.0553 (0.03–0.08)**	**0.0534 (0.03–0.08)**	**0.0523 (0.03–0.08)**	
High viral load	0.0219 (-0.05-0.10)	0.0438 (-0.05-0.13)	0.0532 (-0.04-0.14)	0.0480 (-0.04-0.14)	0.0528 (-0.04-0.14)	0.0614 (-0.03-0.15)	0.0666 (-0.02-0.16)	0.0652 (-0.03-0.16)	0.0652 (-0.03-0.16)	0.0652 (-0.03-0.16)	0.0652 (-0.03-0.16)	
Low-level viremia	-0.0033 (-0.00-0.00)	-0.0266 (-0.04-0.01)	**-0.0571 (-0.09–0.03)**	**-0.0803 (-0.11–0.05)**	**-0.0921 (-0.13–0.06)**	**-0.1000 (-0.14–0.06)**	**-0.1068 (-0.14–0.07)**	**-0.1113 (-0.15–0.08)**	**-0.1113 (-0.15–0.08)**	**-0.1113 (-0.15–0.08)**	**-0.1113 (-0.15–0.08)**	
Country												
Kenya	-0.0043 (-0.01-0.00)	-0.0065 (-0.01-0.00)	-0.0071 (-0.02-0.00)	-0.0085 (-0.02-0.00)	-0.0100 (-0.02-0.00)	-0.0090 (-0.02-0.00)	-0.0002 (-0.02-0.02)	0.0123 (-0.01-0.03)	0.0218 (-0.00-0.05)	**0.0241 (0.00-0.05)**	**0.0241 (0.00-0.05)**	
Uganda	0.0069 (-0.07-0.09)	-0.0117 (-0.09-0.07)	-0.0233 (-0.10-0.06)	-0.0342 (-0.12-0.05)	-0.0393 (-0.12-0.04)	0.0707 (-0.09-0.23)	0.0676 (-0.10-0.23)	0.0655 (-0.10-0.23)	0.0612 (-0.10-0.22)	0.0548 (-0.11-0.22)	0.0512 (-0.11-0.21)	
Tanzania	**0.0265 (-0.00-0.06)**	**0.0604 (0.01–0.11)**	**0.0528 (0.00-0.10)**	0.0489 (-0.00-0.10)	0.0489 (-0.00-0.10)	0.0489 (-0.00-0.10)	0.0489 (-0.00-0.10)	0.0489 (-0.00-0.10)	0.0489 (-0.00-0.10)	0.0489 (-0.00-0.10)	0.0489 (-0.00-0.10)	

Values represent excess cumulative mortality (hypertensive minus non-hypertensive) expressed as probabilities with 95% confidence intervals in parentheses. Positive values indicate higher mortality among hypertensives. Bold indicates estimates where the confidence interval excludes zero (statistically significant at α = 0.05). Intervals formatted as “-0.00” represent values that round to zero from a negative number; these intervals include zero and are therefore not statistically significant. “0.00” are a small positive number rounded to zero therefore significant. Early intervals (Years 1–3) contain sparse data; estimates from these periods should be interpreted with caution. Values in bold indicate statistical significance (*p* < 0.05)

### Multivariable results

**Fig. 1:**
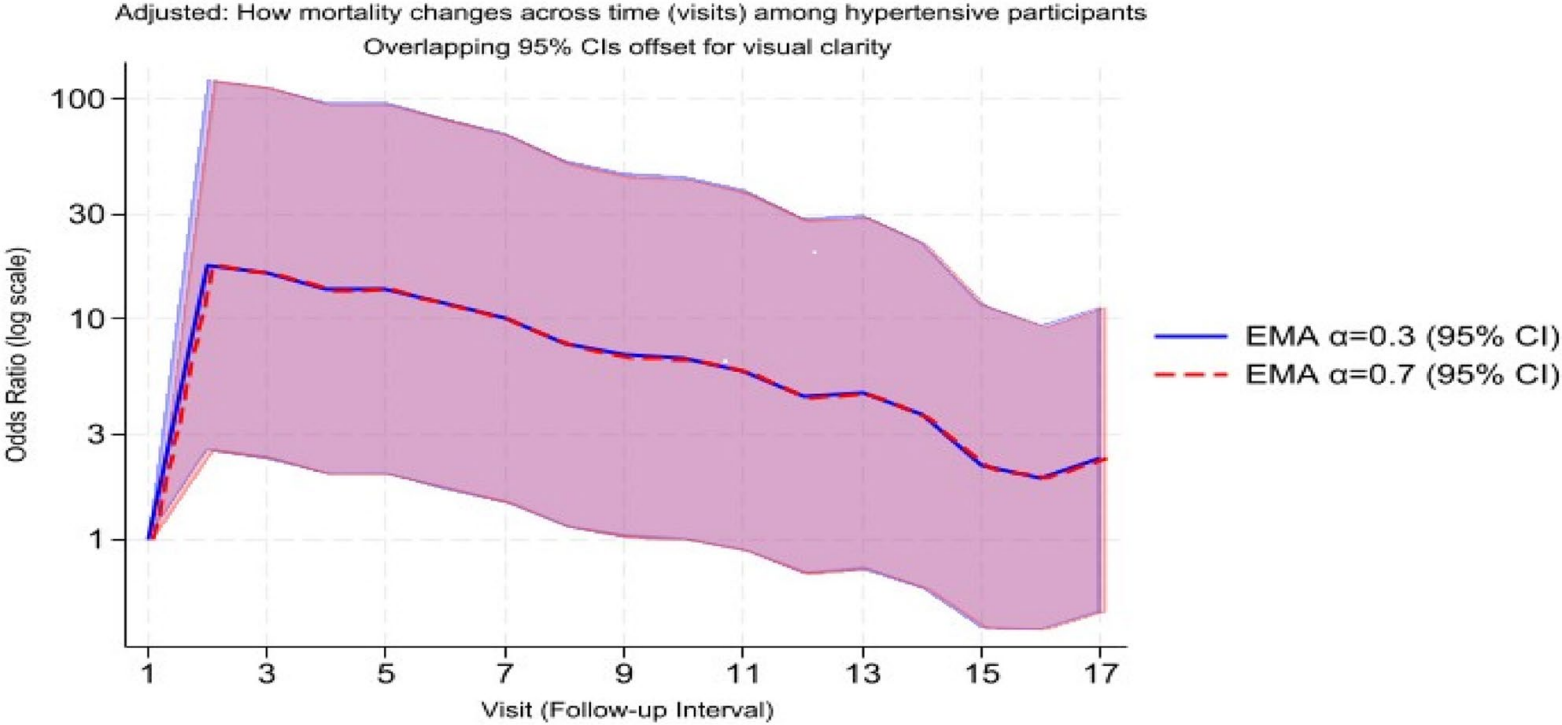
Adjusted temporal pattern of mortality among hypertensive participants under alternative exposure-weighting schemes (EMA α = 0.3 and α = 0.7)

Figure 1 presents the odds ratio (log scale) for all-cause mortality associated with hypertension across successive follow-up visits, estimated from adjusted discrete-time logistic regression models. Shaded bands indicate 95% confidence intervals. Both models reveal elevated mortality risk during the early observation period (visits 2–7; OR ≈ 13–18), followed by progressive attenuation to below OR = 3 by visit 16. The close alignment of the two curves demonstrates the robustness of the results to alternative smoothing parameters: α = 0.3 captures long-term cumulative exposure, while α = 0.7 emphasizes short-term variation.

Discrete-time logistic regression models (Table 4) quantified mortality differentials associated with hypertension after accounting for demographic and clinical composition. Hypertension was associated with a two- to threefold increase in the odds of death, with consistent estimates across exposure specifications: standard lag model (AOR = 2.04; 95% CI 1.10–3.80), EWMA α = 0.3 (AOR = 3.25; 95% CI 1.26–8.40), and EWMA α = 0.7 (AOR = 2.51; 95% CI 1.16–5.44). Compared to virally suppressed individuals, those with low-level viremia have more than double the odds of mortality across exposure specifications; standard lag model (AOR = 2.41; 95% CI 1.16–4.99), EWMA α = 0.3 (AOR = 2.43; 95% CI 1.16–5.09), and EWMA α = 0.7 (AOR = 2.42; 95% CI 1.16–5.04) and those with high viral load; standard lag model (AOR = 2.36 95% CI 1.28–4.33) EWMA α = 0.3 (AOR = 2.44; 95% CI 1.32–4.51), and EWMA α = 0.7 (AOR = 2.39; 95% CI 1.30–4.41) had consistently elevated risk across all model specifications. Body mass index showed a strong inverse association with mortality. Overweight participants had approximately 70% lower odds of death (AOR range: 0.29–0.31; 95% CI ranges: 0.10–0.90), while obese participants had approximately 85% lower odds (AOR range: 0.14–0.15; 95% CI ranges: 0.04–0.59) compared to underweight individuals. Geographic differences were also evident. Mortality risk in Tanzania was approximately 80% lower than in Kenya (AOR range: 0.19–0.21; 95% CI ranges: 0.07–0.55). No significant differences in mortality were observed between Uganda and Kenya or by sex.

**Table 4:** Discrete-time logistic regression of all-cause mortality among PLWH (Unadjusted and adjusted models, AFRICOS 2013–2023)

Predictor	Unadjusted EWMA α = 0.3 OR (95% CI)	Adjusted Standard Lag aOR (95% CI)	Adjusted EWMA α = 0.3 aOR (95% CI)	Adjusted EWMA α = 0.7 aOR (95% CI)
Hypertension exposure	2.15 (0.86–5.33)	**2.04 (1.10–3.80)**	**3.25 (1.26–8.40)**	**2.51 (1.16–5.44)**
Country (ref = Kenya)				
Tanzania	—	**0.21(0.08–0.55)**	**0.19 (0.07–0.52)**	**0.20 (0.08–0.54)**
Uganda	—	1.21 (0.52–2.82)	1.27 (0.54–2.96)	1.24 (0.53–2.89)
Age in years (ref = 40–49)				
50–59	—	1.16 (0.59–2.27)	1.10 (0.56–2.16)	1.13 (0.57–2.23)
60 +	—	2.40 (0.94–6.16)	2.23 (0.85–5.83)	2.32 (0.89–6.01)
Sex (ref = female)		0.53 (0.24–1.17)	0.63 (0.31–1.25)	0.63 (0.31–1.26)
BMI (kg/m²) (ref = underweight [< 18.5])				
Normal (18.5–24.9)	—	0.63 (0.31–1.31)	0.63 (0.30–1.30)	0.63 (0.31–1.31)
Overweight (25.0–29.9)	—	**0.31 (0.11–0.90)**	**0.29 (0.10–0.85)**	**0.30 (0.10–0.88)**
Obese (≥ 30.0)	—	**0.15(0.04–0.59)**	**0.14 (0.04–0.53)**	**0.14 (0.04–0.56)**
Viral load (copies/mL) (ref = suppressed [< 50])				
Low viremia (50–999)	—	**2.41 (1.16–4.99)**	**2.43 (1.16–5.09)**	**2.42 (1.16–5.04)**
High viral load (≥ 1000)	—	**2.36 (1.28–4.33)**	**2.44 (1.32–4.51)**	**2.39 (1.30–4.41)**
Observations (person-periods)	12 462	12 462	12 462	12 462

Values in bold indicates statistical significance (*p*< 0.05).* OR *odds ratio*, aOR *adjusted odds ratio,* CI *confidence interval,* EWMA *Exponentially Weighted Moving Average (exposure measure), smoothing parameter. All adjusted models include time fixed effects for follow-up wave. BMI-categories are based on WHO classification

## Discussion

The objective of this study was to examine how hypertension is associated with all-cause mortality trajectories among people living with HIV (PLWH) aged 40 years and above in Kenya, Uganda, and Tanzania. Life-table analyses address cumulative mortality experience at the population level and revealed widening excess mortality among hypertensive participants over follow-up, particularly among older adults, men, and underweight individuals. These patterns capture the accumulation of unadjusted mortality differences as cohorts age under sustained ART.

By contrast, discrete-time logistic regression models estimate conditional mortality after accounting for measured demographic and clinical characteristics. In these adjusted models, hypertension remained strongly associated with mortality, while sex and age group exhibited attenuated or non-significant effects. This pattern reflected the distinction between unconditional, cumulative differences (life tables) and conditional, composition-adjusted effects (regression). The covariates that appear salient in life tables often share pathways or exposure histories, so adjustment for confounding reduces their independent contribution. Together, the two approaches demonstrate that hypertension is an independent mortality risk embedded within a survival-selected population, while other apparent differentials largely reflect selective survival and compositional change.

Context within Demographic Transition Theory: The epidemiologic transition framework [30] describes population-level shifts from infectious to chronic disease mortality over historical time. Our findings suggest a similar transition occurring within a defined subpopulation over a single decade, driven not by socioeconomic development but by biomedical intervention (ART). This may be a “micro-epidemiologic transition” where the compression of mortality has shifted into a single treatment generation. This has been hypothesized but rarely quantified using demographic methods [30]. The life-table approach is uniquely suited to capture this phenomenon because it summarizes cumulative mortality experience across successive intervals, revealing how risk redistributes as cohorts age.

The attenuation of excess mortality at older ages (among participants aged 60+) is consistent with classic demographic processes of selective survival and frailty redistribution [31]. Under sustained ART, frailer individuals are removed from the cohort earlier, leaving a progressively more robust survivor population. This compositional change seems not a diminution of hypertension’s biological effect but an explanation of why mortality differentials narrow at advanced ages. The inverse BMI-mortality gradient we observed (highest excess among underweight participants) further illustrates this process: underweight status in this population likely is consistent with advanced disease progression or comorbidity burden, marking individuals for earlier death [32]. Life tables capture how such compositional shifts reshape the mortality profile of an ageing cohort over time.

Similarly, direct cardiovascular pathway may be in operation whereby hypertension contributes to mortality through accelerated atherosclerosis and end-organ damage, consistent with studies linking HIV infection and long-term ART exposure to endothelial dysfunction and metabolic dysregulation [33–35]. However, without cause-specific mortality data, this mechanism remains speculative. The multimorbidity pathway may be involved, where hypertension serves as a sentinel for broader chronic disease burden. Hypertensive individuals may be more likely to have undiagnosed diabetes, renal impairment, or other comorbidities that independently increase mortality. This interpretation is supported by the clustering of cardiovascular risk factors observed in aging PLHIV cohorts [33–35].

The pronounced excess mortality among men and its absence among women until late follow-up is consistent with well-documented sex differentials in survival and healthcare engagement [36]. In demographic terms, male mortality disadvantage persists even within a treatment-saturated population, suggesting that the forces governing sex differences in survival operate independently of HIV care. Country-level variation where Tanzania showing earlier and more persistent excess than Kenya or Uganda likely is consistent with differences in the timing and pace of ART scale-up, producing staggered cohort aging across the region [15]. These national differentials illustrate how health-system factors interact with demographic processes to shape mortality outcomes.

Hypertension’s higher prevalence among virally suppressed individuals underscores its role not as treatment failure but as a marker of successful survival through the HIV care cascade. Research from Tanzania and Cameroon similarly report that suppressed viral load is paradoxically associated with higher hypertension risk, reflecting that patients with better immune recovery survive longer, allowing age-related conditions to emerge [33, 37]. Those who remain engaged in care long enough to develop hypertension have, by definition, have likely survived the period of highest infectious mortality risk. Hypertension thus indexes the shifting composition of the cohort itself signalling a demographic signature of the transition from an infectious to a chronic mortality regime. Life tables revealed this process directly by showing how excess mortality emerges and widens as the cohort ages, tracking the accumulation of chronic disease burden among survivors.

### Limitations

Several limitations warrant consideration. First, temporal ambiguity persists as precise dates for HIV diagnosis, ART initiation, and hypertension onset were unavailable, limiting our ability to establish definitive causal sequencing. Second, selective survival may influence age-specific estimates. The attenuation of mortality differences at older ages likely reflects that frailer individuals die earlier, leaving a more robust survivor population, rather than a true diminution of hypertension’s effect [31]. Third, residual confounding cannot be excluded. Unmeasured factors (socioeconomic status, health behaviors, healthcare access, medication adherence) may influence both hypertension risk and mortality. Fourth, selection bias is inherent to clinic-based cohorts; our sample may not represent PLHIV lost to follow-up or never diagnosed. Fifth, measurement error may affect findings. Blood pressure was measured at study visits rather than through standardized protocols, and cause-of-death data relied on study-specific codes rather than ICD-10 classification, with 58.1% of deaths coded as unknown. This under-ascertainment of cardiovascular deaths (5.4%) precludes cause-specific decomposition. Sixth, reverse causality is a concern. A subset of participants with intermittent hypertension exhibited disproportionately high mortality (12.8 per 1000 PY), suggesting blood pressure fluctuations may reflect acute illness rather than chronic hypertension. While our primary analysis classified these individuals as exposed, sensitivity analyses requiring persistent hypertension produced implausible negative excess mortality estimates, confirming that such an approach would misclassify high-risk individuals and artificially bias comparisons. Future studies with more frequent BP monitoring and data on acute illness episodes are needed to distinguish transient from sustained hypertension.

### Implications for demographic research

This study demonstrates the value of decremental life tables for understanding how chronic diseases reshape mortality within aging HIV cohorts. As ART creates unprecedented survivorship among previously high-mortality populations, demographers have an opportunity to observe compressed transitions that mirror, in miniature, the historical shifts described by Omran and others. Future research should build on this foundation by applying formal frailty models to quantify how selective survival redistributes risk, linking clinical cohorts with verbal autopsy or vital registration data to enable cause-specific decomposition, and extending life-table analysis to additional chronic conditions such as diabetes and renal disease. Such work would require moving beyond the descriptive demographic question to the causal question of how ART duration, adherence, and regimen interact with hypertension trajectories to shape survival.

## Conclusion

The mortality experience of people living with HIV in East Africa has entered a new demographic phase in which hypertension plays a central role. As antiretroviral therapy extends survival into older ages, mortality is increasingly shaped by chronic vulnerabilities of longevity rather than infectious causes alone. Hypertension appears as both a driver of excess mortality and a marker of selective survival, reflecting the accumulation of chronic disease risk among long-term ART users. The AFRICOS cohort reveals a redistribution of frailty toward older, heavier, and predominantly female survivors, signaling structural ageing of the treated HIV population. These findings provide evidence of a micro-epidemiologic transition within HIV cohorts and underscore the need to integrate hypertension and chronic disease management into long-term HIV care.

## Data Availability

The datasets analysed during the current study are not publicly available due to privacy protections but are available from the corresponding author on reasonable request. The Henry M. Jackson Foundation for the Advancement of Military Medicine (HJF) and the Walter Reed Army Institute of Research (WRAIR) are committed to safeguarding the privacy of research participants. The distribution of data will require compliance with all applicable regulatory and ethical processes, including the establishment and approval of an appropriate data-sharing agreement. To request a minimal dataset, please contact the data coordinating and analysis center (DCAC) at PubRequest@hivresearch.org along with the name of this manuscript. The authors confirm that they have permission from the relevant institutions to make data available through this process.

## Abbreviations

AFRICOS: African Cohort Study
aOR: Adjusted Odds Ratio
ART: Antiretroviral Therapy
BMI: Body Mass Index
CI: Confidence Interval
CRVS: Civil Registration and Vital Statistics
DCAC: Data coordinating and analysis centre
EWMA: Exponentially Weighted Moving Average
HIV: Human Immunodeficiency Virus
HJF: Henry M. Jackson Foundation for the Advancement of Military Medicine
NCDs: Non-Communicable Diseases
OR: Odds Ratio
PLWH: People Living with HIV
REC: Research Ethics Committee
WRAIR: Walter Reed Army Institute of Research
Δqₓ: Difference in probability of death (excess mortality)

## References

[1] World Health Organisation. Noncommunicable Diseases. 2024. Retrieved 08/01/2026. https://www.afro.who.int/health-topics/noncommunicable-diseases.

[2] Barry A, et al. Non-communicable diseases in the WHO African region: analysis of risk factors, mortality, and responses based on WHO data. Sci Rep. 2025;15(1):12288. 40210980 10.1038/s41598-025-97180-3 PMC11986008

[3] World Health Organisation. Communicable and non-communicable diseases in Africa in 2021/22. 2024. https://www.afro.who.int/sites/default/files/2023-08/Disease%20outlook%20report_BLF_revised_190823_AHN.pdf.

[4] Chen A, et al. Hypertension among people living with human immunodeficiency virus in sub-Saharan Africa: a systematic review and meta-analysis. Sci Rep. 2024;14(1):16858. 39039244 10.1038/s41598-024-67703-5 PMC11263367

[5] Patel P, et al. Noncommunicable diseases among HIV-infected persons in low-income and middle-income countries: a systematic review and meta-analysis. Aids. 2018;32(1):S5–20. 29952786 10.1097/QAD.0000000000001888 PMC6380891

[6] Negin J, et al. Aging with HIV in Africa: the challenges of living longer. Aids. 2012;26(0 1):S1–5. 22713477 10.1097/QAD.0b013e3283560f54 PMC4017661

[7] Reniers G, et al. Mortality trends in the era of antiretroviral therapy: evidence from the Network for Analysing Longitudinal Population based HIV/AIDS data on Africa (ALPHA). Aids. 2014;28(Suppl 4):S533–42. 25406756 10.1097/QAD.0000000000000496 PMC4251911

[8] Nabukalu D, et al. Population-level adult mortality following the expansion of antiretroviral therapy in Rakai, Uganda. Popul Stud (Camb). 2020;74(1):93–102. 31117928 10.1080/00324728.2019.1595099 PMC6891159

[9] Autenrieth CS, et al. Global and regional trends of people living with HIV aged 50 and over: Estimates and projections for 2000–2020. PLoS ONE. 2018;13(11):e0207005. 30496302 10.1371/journal.pone.0207005 PMC6264840

[10] Moyo-Chilufya M, et al. The burden of non-communicable diseases among people living with HIV in Sub-Saharan Africa: a systematic review and meta-analysis. EClinicalMedicine. 2023;65:102255. 37842552 10.1016/j.eclinm.2023.102255 PMC10570719

[11] Smit M, et al. Future challenges for clinical care of an ageing population infected with HIV: a modelling study. Lancet Infect Dis. 2015;15(7):810–8. 26070969 10.1016/S1473-3099(15)00056-0 PMC4528076

[12] Kaluvu L, et al. Multimorbidity of communicable and non-communicable diseases in low- and middle-income countries: A systematic review. J Multimorb Comorb. 2022;12:26335565221112593. 36081708 10.1177/26335565221112593 PMC9445468

[13] Tegegne KD, et al. Prevalence and factors associated with hypertension among peoples living with HIV in East Africa, a systematic review and meta-analysis. BMC Infect Dis. 2023;23(1):724. 37880643 10.1186/s12879-023-08679-x PMC10601241

[14] Masenga SK, et al. Patho-immune Mechanisms of Hypertension in HIV: a Systematic and Thematic Review. Curr Hypertens Rep. 2019;21(7):56. 31165257 10.1007/s11906-019-0956-5 PMC6548744

[15] Mollel GJ, et al. Causes of death and associated factors over a decade of follow-up in a cohort of people living with HIV in rural Tanzania. BMC Infect Dis. 2022;22(1):37. 34991496 10.1186/s12879-021-06962-3 PMC8739638

[16] Chidumwa G, Mazibuko L, Olivier S, Rahman K, Gareta D, Aung TN, Busang J, Herbst K, Wong E, Baisley K. HIV, hypertension and diabetes care and all-cause mortality in rural South Africa in the HIV antiretroviral therapy era: a longitudinal cohort study. BMJ Public Health. 2023;1(1). 10.1136/bmjph-2023-000153.

[17] Kuate Defo B. Demographic, epidemiological, and health transitions: are they relevant to population health patterns in Africa? Glob Health Action. 2014;7:22443. 24848648 10.3402/gha.v7.22443 PMC4028929

[18] Beltrán-Sánchez H, Preston SH, Canudas-Romo V. An integrated approach to cause-of-death analysis: cause-deleted life tables and decompositions of life expectancy. Demogr Res. 2008;19:1323. 20165568 10.4054/DemRes.2008.19.35 PMC2822405

[19] Dannefer D. Cumulative advantage/disadvantage and the life course: cross-fertilizing age and social science theory. J Gerontol B Psychol Sci Soc Sci. 2003;58(6):S327–37. 14614120 10.1093/geronb/58.6.s327

[20] Elder GH, Shanahan MJ. The life course and human development. Handb Child Psychol. 2007;1. 10.1002/9780470147658.chpsy0112.

[21] McEwen BS. Stress, adaptation, and disease. Allostasis and allostatic load. Ann N Y Acad Sci. 1998;840:33–44. 10.1111/j.1749-6632.1998.tb09546.x. 10.1111/j.1749-6632.1998.tb09546.x 9629234

[22] Ferraro KF, Shippee TP. Aging and cumulative inequality: how does inequality get under the skin? Gerontologist. 2009;49(3):333–43. 10.1093/geront/gnp034 19377044 10.1093/geront/gnp034 PMC2721665

[23] Chang D, et al. Non-communicable diseases in older people living with HIV in four African countries: a cohort study. Lancet HIV. 2022;9:S5.

[24] Bigna JJ, et al. Global burden of hypertension among people living with HIV in the era of increased life expectancy: a systematic review and meta-analysis. J Hypertens. 2020;38(9):1659–68. 32371769 10.1097/HJH.0000000000002446

[25] Bloomfield GS, et al. Blood pressure level impacts risk of death among HIV seropositive adults in Kenya: a retrospective analysis of electronic health records. BMC Infect Dis. 2014;14:1–10. 24886474 10.1186/1471-2334-14-284 PMC4046023

[26] Connelly PJ, Currie G, Delles C. Sex Differences in the Prevalence, Outcomes and Management of Hypertension. Curr Hypertens Rep. 2022;24(6):185–92. 35254589 10.1007/s11906-022-01183-8 PMC9239955

[27] Allison P. Fixed Effects Regression Models. Thousand Oaks, California: SAGE Publications, Inc.; 2009.

[28] Smit AC, Schat E, Ceulemans E. The Exponentially Weighted Moving Average Procedure for Detecting Changes in Intensive Longitudinal Data in Psychological Research in Real-Time: A Tutorial Showcasing Potential Applications. Assessment. 2023;30(5):1354–68. 35603660 10.1177/10731911221086985 PMC10248291

[29] Colin Cameron A, Miller DL. A Practitioner’s Guide to Cluster-Robust Inference. J Hum Resour. 2015;50(2):317–72.

[30] Omram AR. The epidemiologic transition: a theory of the epidemiology of population change. Bull World Health Organ. 2001;79(2):161–70. PMC2566347 11246833

[31] Vaupel JW, Manton KG, Stallard E. The impact of heterogeneity in individual frailty on the dynamics of mortality. Demography. 1979;16:439–54. 510638

[32] Bannister WP, et al. Changes in body mass index and clinical outcomes after initiation of contemporary antiretroviral regimens. AIDS. 2022;36(15):2107–19. 35848573 10.1097/QAD.0000000000003332

[33] Kato I, Tumaini B, Pallangyo K. Prevalence of non-communicable diseases among individuals with HIV infection by antiretroviral therapy status in Dar es Salaam, Tanzania. PLoS ONE. 2020;15(7):e0235542. 32645054 10.1371/journal.pone.0235542 PMC7347196

[34] Madhur MS, et al. Hypertension: do inflammation and immunity hold the key to solving this epidemic? Circul Res. 2021;128(7):908–33. 10.1161/CIRCRESAHA.121.318052 PMC8023750 33793336

[35] Masenga SK, et al. Hypertension and metabolic syndrome in persons with HIV. Curr Hypertens Rep. 2020;22:1–8. 32880756 10.1007/s11906-020-01089-3 PMC7467859

[36] Fernandez D, et al. Assessing sex differences in viral load suppression and reported deaths using routinely collected program data from PEPFAR-supported countries in sub-Saharan Africa*.* BMC Public Health. 2023;23(1):1941. 10.1186/s12889-023-16453-6. 10.1186/s12889-023-16453-6 PMC10559393 37805465

[37] Kouanfack C, et al. Hypertension Burden and Care Cascade Gaps Among People Living With HIV in an Urban HIV Clinic in Cameroon 2024. J Int Association Providers AIDS Care (JIAPAC). 2025;24:23259582251378538. 10.1177/23259582251378538 PMC12437170 40953001

